# Determinants of workplace perceptions among federal, state, and local public health staff in the US, 2014 to 2017

**DOI:** 10.1186/s12889-021-11703-x

**Published:** 2021-09-10

**Authors:** Jonathon P. Leider, Katie Sellers, Jessica Owens-Young, Grace Guerrero-Ramirez, Kyle Bogaert, Moriah Gendelman, Brian C. Castrucci

**Affiliations:** 1 University of Minnesota School of Public Health, D312 Mayo Building, MMC 729, 420 Delaware St. SE, Minneapolis, MN 55455 USA; 2 de Beaumont Foundation, 7501 Wisconsin Avenue, Suite 1310e, Bethesda, MD 20814 USA; 3 American University, 4400 Massachusetts Avenue NW, Washington DC, 20016 USA; 4 Association of State and Territorial Health Officials, 2231 Crystal Drive, Suite 450, Arlington, VA 22202 USA

**Keywords:** Public health workforce, Job satisfaction, Intent to leave, Workplace perceptions, Public health workforce interests and needs survey

## Abstract

**Background:**

The governmental public health workforce in the United States comprises almost 300,000 staff at federal, state, and local levels. The workforce is poised for generational change, experiencing significant levels of retirement. However, intent to leave for other reasons is also substantial, and diversity is lacking in the workforce.

**Methods:**

Workforce perception data from 76,000 staff from Health and Human Services (HHS) including 14,000 from the Centers for Disease Control and Prevention were analyzed across 2014 and 2017. Additionally, data from 32,000 state and local health department staff in 46 agencies reporting in both years. Estimates were constructed accounting for survey design and non-response.

**Results:**

In 2017, women made up 43% of the total US government workforce and 33% of supervisors or higher, compared to 73 and 68% generally in State Health Agencies (*p* < .0001); and 62% vs 52% in HHS (*p* < .0001). Among state staff, intent to leave increased from 22 to 31% (*p* < .0001), but fell in 2017 from 33 to 28% for HHS (*p* < .0001). Correlates of intent to leave included low job satisfaction, pay satisfaction, and agency type. Federal entities saw the highest proportion respondents that indicated they would recommend their organization as a good place to work.

**Conclusions:**

While intent to leave fell at federal agencies from 2014 to 2017, it increased among staff in state and local health departments. Additionally, while public health is more diverse than the US government overall, significant underrepresentation is observed in supervisory positions for staff of color, especially women.

**Supplementary Information:**

The online version contains supplementary material available at 10.1186/s12889-021-11703-x.

## Background

A strong, satisfied, and diverse governmental public health agency workforce is critical to promoting good health and responding effectively to the emergent and pressing health and safety needs of our population. Yet, governmental public health agencies in the United States operate amid steep budget cuts [1] and a shrinking workforce [2]. A study [3] using the 2014 Public Health Workforce Interests and Needs Survey (PH WINS) and the Federal Employee Viewpoint Survey (FEVS) found that about 40% of local, state, and federal workers were considering departing their organization or planning to retire by 2020. Estimates suggest that between 2016 and 2020, up to 100,000 staff would leave their organizations if the expected retirements occurred [4]. The high proportion of staff who intend to depart their organizations coupled with an aging workforce [4] raises concerns about the looming threat of workforce turnover.

Voluntary turnover is costly, partly due to the costs associated with the recruitment and training of new and competent staff [5]. Estimates show that turnover may amount to approximately 50 to 200% of a worker’s annual salary in the private sector [6]. Lack of funding may lead to prolonged job vacancies or hiring at a lower pay or educational level [7]. Conversely, retention of skilled staff facilitates the preservation and transfer of institutional knowledge and may alleviate the burden associated with recruitment [5].

Employee satisfaction and perceptions and opinions about one’s workplace environment (“workplace perceptions”) are associated with staff’s intentions to leave and turnover [8–10]. Therefore, identifying the factors associated with job satisfaction and positive workplace perceptions can inform strategies that are conducive to the retention and recruitment of skilled staff at public health agencies, which include administrative/clerical, public health science, clinical, laboratory, and other social services staff [3].

In addition, successful diversity and inclusion management is a cornerstone of a robust workforce. Given that non-Hispanic White employees make up the majority of the governmental public health workforce [3], it is important to explore how intent to leave and perceptions about the workplace may differ across racial and ethnic groups. For instance, a prior study focusing on state health agencies and local health departments found that the odds of considering leaving were 30% higher for staff of color compared to non-Hispanic White staff [11].

Previous analyses have used PH WINS to compare demographic variables and workplace-related outcomes between 2014 and 2017, though not for federal employees [11, 12]. Using PH WINS and FEVS, the aim of this article is to assess and compare employee satisfaction, workplace perceptions, intent to leave, and demographic variables among federal, state, and local public health staff in the US between 2014 and 2017.

## Methods

This manuscript characterizes changes in workplace perception, job satisfaction, and intent to leave among governmental public health workers in the US, between 2014 and 2017. It does so using two primary datasets, the Federal Employee Viewpoint Survey (FEVS) and the Public Health Workforce Interests and Needs Survey (PH WINS). FEVS has been conducted since 2002, and focuses on the federal workforce [13]. It includes a number of workplace perception questions and demographic characteristics. The survey is anonymized, so it is functionally a multi cross sectional survey when used over multiple years. Simple nonresponse adjustment is conducted by agency and subunits within agencies. FEVS heavily influenced the development of PH WINS in 2014, with several questions being used directly in the instrument, allowing for comparisons across studies, over time. PH WINS was developed to investigate workplace perceptions, job satisfaction, training needs, awareness of emerging public health concepts, and demographics in the state and local governmental public health workforce.

In summer 2014, FEVS was fielded to 872,495 people, with 47% responding. In summer 2017, FEVS was fielded to 1.1 million people with 45% responding. It is fielded with a graduated probability sample design, where units with fewer than 50 staff receive FEVS as a census, with 51 to 75 staff having a 75% probability sample drawn, with 76 to 150 having a 50% sample drawn, and with more than 150 having a 25% sample drawn. PH WINS has a somewhat different design. In fall 2014, PH WINS was fielded as a nationally representative sample of State Health Agency central offices (SHA-COs), as well as a census of large local health departments. These large local health departments are members of the Big Cities Health Coalition (BCHC), which represents the largest local health departments (LHDs) in the US. PH WINS was also fielded as a pilot to other local health departments in the US. In fall 2017, when PH WINS was fielded again, it was expanded to include a census of SHA-COs and a nationally representative sample of LHDs was added. However, this manuscript attempts to maximize comparability between 2014 and 2017, and thus only agencies that participated in both years are included. For the FEVS, the analytic sample includes the Centers for Disease Control and Prevention (CDC), the Department of Health and Human Services (HHS) in total, and the US government in total. For PH WINS, 33 SHA-COs participated with sufficient responses in both years, as did 13 BCHC LHDs. Taylor series linearization is used to adjust for non-response and sampling design. As shown in previous work [12], this approach has some generalizability to state and local health departments nationally, but should be viewed as a multi cross sectional comparison across participating agencies in 2014 and 2017.

Analysis for this manuscript was conducted in 2020 and focuses on characterization of the change in workplace perceptions between 2014 and 2017, as well as inferential analysis of differentials of those changes. Descriptive statistics are provided. Bivariate comparisons are made using Rao Scott adjusted chi square. In particular, workplace environment, job satisfaction, and intent to leave are examined by gender and race/ethnicity. Representativeness in supervisors compared to staff in general was also compared using a Rao Scott chi square. Finally, a logistic model was fitted, with the dependent variable being whether respondents agreed or strongly agreed that they recommended their organization as a good place to work. Independent variables included gender, race/ethnicity, supervisory status, educational attainment, intent to leave, agency type, year, and several workplace environment variables. A stratified analysis was also conducted by agency type. Variance inflation factor analysis was used to examine collinearity, and survey and non-response weights were applied. Data were managed and analyzed in Stata 15.1 (StataCorp LLC, College Station, Texas), and visualized in Excel (Microsoft, Redmond, WA) and Tableau (Tableau Software, Seattle, WA).

## Results

Across all frames and years, about 911,000 unduplicated responses are included in the analytic sample. This includes 24,474 from SHA-CO, 7585 from BCHC LHDs, 75,892 from HHS including 13,913 from CDC, and a total of 878,857 from the federal government. In 2017, while women made up less than half (43%) of the total US government workforce, they were the majority of the workforce in SHA-COs, BCHC LHDs, CDC, and HHS at 73, 74, 64 and 63%, respectively (Table 1). Between 2014 and 2017, there were increases of people of color (POC) in in SHA-COs from 29% in 2014 to 36% in 2017 (*p* < .0001), BCHC LHDs from 70 to 74% (*p* = .0013), CDC from 43 to 45% (*p* = .0141), and HHS from 50 to 52% (*p* < .0001). However, POC representation decreased in the federal government overall from 37% in 2014 to 36% in 2017 (*p* < .0001) (Appendix Figure 1). Overall, staff had high levels of educational attainment. Among SHA-CO staff in 2017, 41% had a master’s degree. Comparatively, 38% of BCHC staff, 71% of CDC staff, and 33% of all US government staff did. With the exception of BCHC health departments, agencies saw their educational attainment rise between 2014 and 2017.

**Table 1:** Demographics from analytic sample, by agency and year

Agency / Type	Year	Women	People of color	Supervisors or higher	Bachelor’s or higher attainment	Graduate degree attainment
SHA-CO	2014 (*n* = 10,216)	72% (71–73%)	29% (28–30%)	30% (29–31%)	75% (74–76%)	41% (40–42%)
	2017 (*n* = 14,258)	73% (72–74%)	36% (35–37%)	29% (28–30%)	75% (74–76%)	41% (40–42%)
BCHC LHD	2014 (*n* = 2627)	76% (73–78%)	70% (68–72%)	28% (26–30%)	73% (71–75%)	40% (38–43%)
	2017 (*n* = 4958)	74% (73–76%)	74% (73–75%)	27% (26–29%)	73% (72–74%)	38% (36–39%)
CDC (HHS)	2014 (*n* = 6562)	60% (59–61%)	43% (42–44%)	19% (19–20%)	87% (86–88%)	68% (66–69%)
	2017 (*n* = 7351)	64% (63–65%)	45% (44–47%)	18% (17–19%)	91% (90–91%)	71% (70–73%)
HHS	2014 (*n* = 32,806)	62% (61–63%)	50% (49–51%)	19% (18–19%)	77% (76–77%)	53% (53–54%)
	2017 (*n* = 43,086)	63% (62–63%)	52% (51–52%)	17% (17–18%)	80% (80–81%)	58% (57–58%)
US Gov total	2014 (*n* = 392,752)	43% (43–43%)	37% (37–38%)	18% (18–18%)	63% (62–63%)	30% (29–30%)
	2017 (*n* = 486,105)	43% (42–43%)	36% (36–37%)	15% (15–15%)	66% (66–66%)	33% (33–33%)

Cells shown as: Estimate (95% Confidence interval). Thirty three agencies are included in the SHA CO estimates and thirteen BCHC LHDs are. Unweighted response counts are reported by year

*SHA-CO* State Health Agency Central office, *BCHC* Big City Health Department, *CDC* Centers for Disease Control and Prevention, *HHS* Health and Human Services, *US Gov total* all federal employees of US government

**Fig. 1:**
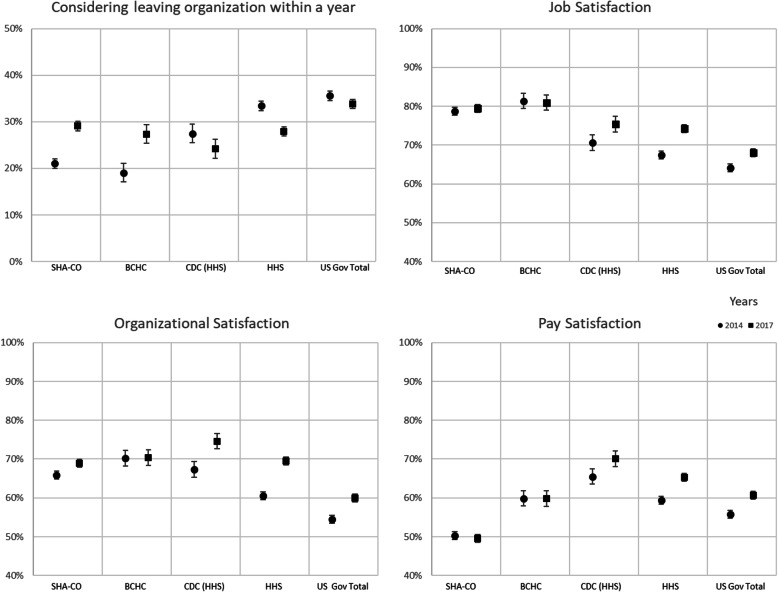
Satisfaction and intent to leave, 2014–2017 by organization type. Notes: SHA-CO – State Health Agency Central office; BCHC – Big City Health Department; CDC – Centers for Disease Control and Prevention; HHS – Health and Human Services; US Gov total – all federal employees of US government. Bars are 95% confidence intervals

Job satisfaction was highest among SHA-CO and BCHC staff (79 and 81%, respectively (Fig. 1), Appendix Figures 2 and 3). Job satisfaction increased from 71 to 75% at CDC from 2014 to 2017 (*p* < .0001), and from 64 to 68% in the US government overall (*p* < .0001). Organizational satisfaction was highest at CDC (75%). Pay satisfaction was highest at CDC (70%), and lowest among SHA-CO staff (50%) in 2017. While pay satisfaction increased across the various federal agencies in the analytic sample, it was unchanged at SHA-CO and LHDs between 2014 and 2017. Intent to leave varied considerably across the agency types. Among SHA-CO staff and BCHC staff, intent to leave increased from 22 to 31% (*p* < .0001) and 20 to 29% (*p* < .0001) from 2014 to 2017, respectively. Among federal agencies in the sample, intent to leave for reasons other than retirement fell in 2017 from 27 24% for CDC (*p* < .0001), from 33 to 28% for HHS (*p* < .0001), and from 36 to 34% for the US government overall (*p* < .0001).

Significant differences among gender and race/ethnicity variables were observed for supervisory status and workplace perceptions (Table 2 and Appendix Table 1). Uniformly across agencies, women make up a smaller proportion of supervisors, managers, and executives than they do of the entire staff. In SHA-CO, for instance, 68% of supervisors and higher are women (*p* < .0001), while women make up about 73% of staff generally, representing a 7% gap. This gap is the smallest in BCHC LHDs (75% vs 73%, *p* = .031), which has only a 3% gap. The gap is about 15% for CDC (62% women staff vs 53% women supervisors) and HHS (62% vs 52%), and is 23% for the US government overall (43% vs 33%). The differences are more substantial for people of color, and are more substantial when the lack of diversity in staff is compared to the population served by public health agencies.

**Table 2:** Workplace perceptions by setting and POC / White respondent in 2017

	SHA-CO	BCHC	CDC (HHS)	HHS	US Gov total
I know how my work relates to the agency’s goals and priorities	89% / 87%^b^	90% / 88%	89% / 87%^a^	89% / 88%^b^	86% / 84%^c^
The work I do is important	94% / 93%	95% / 94%	93% / 91%	93% / 92%^c^	92% / 90%^c^
My training needs are assessed	53% / 52%	55% / 47%^c^	61% / 58%	58% / 59%^a^	57% / 55%^c^
Creativity and innovation are rewarded	43% / 44%	44% / 47%	57% / 59%	50% / 55%^c^	41% / 41%
I recommend my organization as a good place to work	67% / 68%	72% / 68%^a^	80% / 83%^b^	74% / 78%^c^	66% / 67%^c^
My supervisor provides me with opportunities to demonstrate my leadership skills	67% / 69%^a^	67% / 68%	75% / 78%^b^	70% / 77%^c^	66% / 69%^c^
Supervisors in my work unit support employee development	70% / 73%^b^	70% / 71%	77% / 80%^b^	70% / 78%^c^	65% / 69%^c^
My supervisor treats me with respect	83% / 85%^a^	82% / 84%	85% / 89%^c^	81% / 87%^c^	81% / 84%^c^
Supervisors work well with employees of different backgrounds	68% / 74%^c^	71% / 74%^a^	72% / 82%^c^	66% / 80%^c^	64% / 72%^c^
Considering everything, how satisfied are you with your job?	79% / 80%	82% / 81%	76% / 77%	74% / 76%^c^	68% / 69%
Considering everything, how satisfied are you with your pay?	45% / 52%^c^	59% / 63%^a^	68% / 74%^c^	61% / 71%^c^	57% / 63%^c^
Considering everything, how satisfied are you with your organization?	69% / 69%	72% / 69%	75% / 76%	68% / 73%^c^	60% / 60%

Estimates shown as “Person of Color / non-Hispanic white” proportions. Read as, e.g.: 89% of staff of color in State Health Agency Central Offices indicate they agree/strongly agree with the statement “I know how my work relates to the agency’s goals and priorities,” compared to 87% of non-Hispanic white staff, or 45% of staff of color in State Health Agency Central Offices indicate they are somewhat/very satisfied with their pay

*SHA-CO* State Health Agency Central office, *BCHC* Big Cities Health Coalition Health Department, *CDC* Centers for Disease Control and Prevention, *HHS* Health and Human Services, *US Gov* all federal employees of US government

Differences between POC / White staff are statistically significant at: ^a^ - < .05; ^b^ - < .001; ^c^ - < .0001

Strong agency-type effects were observed, with BCHC staff having a 1.15 adjusted odds ratio (AOR) of recommending their organization as a good place to work compared to SHA-CO staff (*p* = .004). Compared to SHA-CO staff, all federal groups had significantly higher adjusted odds of recommending their organization as a good place to work: CDC (3.02, *p* < .0001), HHS total (2.1, *p* < .00011, and US government total (1.7, *p* < .0001). Interactions suggested stratified analyses were appropriate (Table 3). Final models show gender and race/ethnicity effects were not consistent in scale or direction across the agencies. Supervisors were somewhat less likely to recommend their organization as a good place to work, all else equal. Staff considering leaving in the next year were much less likely (AORs between 0.4 and 0.6, p < .0001). Staff who said they knew how their work related to agency’s goals and priorities were more likely to recommend their organization as a good place to work, as were those who felt creativity and innovation were rewarded. Job satisfaction was also highly correlated with likelihood of recommending one’s organization. Staff in 2017 were somewhat more likely to recommend their organization compared to 2014.

**Table 3:** Results of logistic model characterizing associations with agreeing /strongly agreeing with the statement: “I recommend my organization as a good place to work”

	SHA-CO	BCHC	CDC (HHS)	HHS	US Gov total
**Gender**					
Men	ref	Ref	ref	ref	ref
Women	0.8^c^	0.9	1.2^a^	0.9^a^	1^c^
**Race/ethnicity**					
White	ref	ref	ref	ref	ref
POC	1.1	1.4^b^	1	1	1.1^c^
**Supervisory status**					
Non-supervisor	ref	ref	ref	ref	ref
Supervisor / Manager / Executive	0.9	0.8	0.8^a^	0.9^c^	0.9^c^
**Educational attainment**					
Less than bachelors	ref	ref	ref	ref	ref
Bachelors	0.9^b^	0.7^b^	1.3	1.2^c^	1
Graduate	0.8^c^	0.8^a^	1.5^c^	1.5^c^	1^b^
**Considering leaving in next year**					
No	ref	ref	ref	ref	ref
Yes	0.5^c^	0.4^c^	0.4^c^	0.4^3^	0.5^3^
**I know how my work relates to the agency’s goals and priorities**					
Disagree/Strongly Disagree/Neither	ref	ref	ref	ref	ref
Agree/Strongly Agree	2.5^c^	2.6^c^	2^c^	2.1^c^	2.1^c^
**Creativity and innovation are rewarded**					
Disagree/Strongly Disagree/Neither	ref	ref	ref	ref	ref
Agree/Strongly Agree	3.6^c^	3.2^c^	4.1^c^	3.6^c^	3.2^c^
Disagree/Strongly Disagree/Neither	ref	ref	ref	ref	ref
Agree/Strongly Agree	1.3^c^	1.2	1	1.1^b^	1.1^c^
**Supervisors in my work unit support employee development**					
Disagree/Strongly Disagree/Neither	ref	ref	ref	ref	ref
Agree/Strongly Agree	1.9^c^	2.1^c^	1.3^b^	1.5^c^	1.6^c^
**My supervisor treats me with respect**					
Disagree/Strongly Disagree/Neither	ref	ref	ref	ref	ref
Agree/Strongly Agree	1.7^c^	1.8^c^	1.5^c^	1.4^c^	1.4^c^
**Supervisors work well with employees of different backgrounds**					
Disagree/Strongly Disagree/Neither	ref	ref	ref	ref	ref
Agree/Strongly Agree	2.1^c^	2.5^c^	2.6^c^	2.6^c^	2.3^c^
**Considering everything, how satisfied are you with your job?**					
Very dissatisfied/dissatisfied/neither	ref	ref	ref	ref	ref
Satisfied / Very satisfied	4.4^c^	4.4^c^	6^c^	6.2^c^	7.3^c^
**Year**					
2014	ref	ref	ref	ref	ref
2017	1.1^b^	1.2	1.1	1.2^c^	1^b^
Constant	0.1^c^	0.1^c^	0.1^c^	0.1^c^	0.1^c^

Estimates shown are adjusted odds ratios

AORs are statistically significant compared to the reference category at: ^a^ - < .05; ^b^ - < .001; ^c^ - < .0001

## Discussion

Public health staff across the United States are showing high rates of burnout [14], and concomitantly high intention to leave or retire [12]. Retention issues within public health are not new; the so-called ‘silver tsunami’ of retiring baby boomers has been looming for a decade [4]. Coupled with significant cuts to public health in the aftermath of the Great Recession, and now of the COVID-19 pandemic, longevity of the workforce is in question. Data from the Federal Employee Viewpoint Survey and from state and local public health staff in the Public Health Workforce Interests and Needs Survey demonstrate that most governmental public health employees are satisfied with their jobs and that they agree that their organization is a good place to work. These data also show governmental public health employees who understand how their work relates to the agency’s goals and priorities and believe that their supervisor works well with diverse employees are more likely to recommend their organization as a good place to work, in line with research in other fields [12, 15–18].

There is no question that pay satisfaction directly relates to intention to leave, but it is also not the sole driver [12]. It may not be a point of leverage for many cash-strapped health departments, so selective investment in other aspects of the workplace environment may pay more substantial dividends than improving pay bands for competitive positions alone [19]. As more governmental public health employees are set to retire in the next decade [20], public health leaders must consider strategies to attract the next generation of public health professionals to work in all levels of government.

While much of public health practice in the United States relates in some former fashion to a direct service, be it a clinical service or inspection, response to the COVID-19 pandemic has shown with that even a mobilized workforce can largely work remotely. These shifts in time and place flexibility relate to improved perceptions of public sector work outside of public health, and likely within it [21]. The next generation of workers values flexibility, creativity, diversity, equity, and inclusion across the organization [22]. Leadership may then focus both on concretely improving their organization as a desirable place of employment and ensuring that individuals are remunerated, promoted, and recognized in ways that meaningfully promote retention [22].

Racial and ethnic diversity in the governmental public health workforce continues to grow. But there remain significant racial and ethnic differences in the factors associated with employees’ recommendation of their organization as a good place to work. Race and ethnicity shapes how employees perceive their workplace experiences [23], and while progress has been made in diversifying the governmental public health workforce; people of color remain underrepresented, especially in supervisory and leadership positions [24]. Moreover, staff of color are, on average, paid less than their white peers [25, 26], which may partially account for racial differences in pay satisfaction. A diverse and representative governmental public health workforce may lead to greater health improvements among marginalized populations [27, 28] and contribute to greater gains toward achieving goals of Public Health 3.0, including addressing the social determinants of health [29, 30].

One area of particular interest was in the lack of observed differences in perceptions by race/ethnicity in Big City health departments; there were no significant differences between white staff and staff of color in their responses to “my supervisor treats me with respect,” “supervisors in my work unit support employee development,” and “my supervisor provides me with opportunities to demonstrate my leadership skills.” Supervisors in BCHC organizations are more likely to be women or people of color compared to other agencies, as is the overall workforce in BCHC agencies compared to other governmental public health agencies. This racial and ethnic representation in supervisory positions may contribute to the lack of racial or ethnic differences in perceptions of supervisory support, respect, and leadership opportunities. Supporting racial and ethnic diversity in supervisory roles in the governmental public health workforce may foster environments where staff of color feel not only more represented, but also more supported in their leadership development and aspirations.

Beyond job satisfaction lies more challenging existential questions about the future of governmental public health. Chronic underinvestment in public health systems contributed to the US having some of the worst outcomes in the COVID pandemic [31]. In large part, this is because dollars in public health overwhelmingly go toward people. And in the years leading up to COVID-19, over 50,000 jobs were lost among state and local health departments. To ‘build back better,’ governments across the United States must successfully hire tens of thousands of young people and retain them. This is no small task, and one public health has not historically been successful at [32]. Yet data across two major national surveys gives hints of what leadership in agencies may do - certainly, try and compete on pay where possible, but also invest in improving the workplace environment and creating clear paths for promotion and incentives for retention. Data in these surveys show it may be helpful for all staff, and especially for young and educated staff, who are more likely to be diverse than their older counterparts.

### Limitations

There are several limitations to this study worth considering. The most significant is of generalizability. While it is the case that the FEVS data are representative nationally for 2014 and 2017, we included only SHA-CO and BCHC LHDs that participated in 2014 and 2017. While this increases comparability in a multi-cross sectional approach, it moderates generalizability to non-participating agencies. It is also not representative of mid-size or small LHDs. An additional consideration is the self-reported nature of the results. Because this analysis is generally concerned with workplace perceptions, job satisfaction, and intent to leave, self-report is an appropriate data collection modality. Finally, intent to leave is not completely determinative of actual separations [33]. Research in this population suggests about half of those who intended to leave do, and about a quarter of those who did not indicate they were considering leaving did so between 2014 and 2017.

## Conclusion

While these findings suggest an overall improvement in the governmental public health workplace environment, there is a significant need for agencies to pursue strong retention and recruitment strategies. This involves building employee morale, providing opportunities for growth, and nurturing creativity and innovation. At the same time, it is essential that agencies institutionalize a culture of diversity, equity, and inclusion at all organizational levels.

## Supplementary Information

**Additional file 1: Table 1.** Distribution of supervisory status by gender and race/ethnicity in 2014 & 2017. Note: Shown as Estimate (95% Confidence Interval). **Figure 1.** Percent of staff by supervisory status, by gender and race/ethnicity in 2014 & 2017 (pooled). **Figure 2.** Perceptions of workplace environment. **Figure 3.** Agree/strongly agree with statement: Supervisors work well with employees of different backgrounds. Note: POC – Person of color; White – non-Hispanic White. SHA-CO – State Health Agency Central office; BCHC – Big City Health Department; CDC – Centers for Disease Control and Prevention; HHS – Health and Human Services; EPA – Environmental Production Administration; US Gov total – all federal employees of US government. Bars are 95% confidence interval.

## Data Availability

PH WINS data are available upon request from the de Beaumont Foundation at https://www.phwins.org, and FEVS data are publicly available through the United States Office of Personnel Management at https://www.opm.gov/fevs/public-data-file.

## Abbreviations

PH WINS: Public Health Workforce Interests and Needs Survey
FEVS: Federal Employee Viewpoint Survey
SHA-CO: State Health Agency - Central Office
BCHC: Big City Health Coalition
LHD: Local Health Department
CDC: Centers for Disease Control and Prevention
