# Supplementary material for: Determinants of workplace perceptions among federal, state, and local public health staff in the US, 2014 to 2017
Source: BMC Public Health. 2021 Sep 10;21:1654. doi: 10.1186/s12889-021-11703-x (PMC8431955; doi:10.1186/s12889-021-11703-x)
Supplement: Supplementary file 1 — Additional file 1: Table 1. Distribution of supervisory status by gender and race/ethnicity in 2014 & 2017. Note: Shown as Estimate (95% Confidence Interval). Figure 1. Percent of staff by supervisory status, by gender and race/ethnicity in 2014 & 2017 (pooled). Figure 2. Perceptions of workplace environment. Figure 3. Agree/strongly agree with statement: Supervisors work well with employees of different backgrounds. Note: POC – Person of color; White – non-Hispanic White. SHA-CO – State Health Agency Central office; BCHC – Big City Health Department; CDC – Centers for Disease Control and Prevention; HHS – Health and Human Services; EPA – Environmental Production Administration; US Gov total – all federal employees of US government. Bars are 95% confidence interval. [file 12889_2021_11703_MOESM1_ESM.docx]

Appendix Table 1: Distribution of supervisory status by gender and race/ethnicity in 2014 & 2017

|  | SHA-CO | BCHC | CDC (HHS) | HHS | US Gov total |
| --- | --- | --- | --- | --- | --- |
| Percent of staff that are women | 73% (72%-73%) | 75% (74%-76%) | 62% (61%-63%) | 62% (62%-63%) | 43% (43%-43%) |
| Percent of supervisors or higher that are women | 68% (66%-69%) | 73% (70%-75%) | 53% (51%-55%) | 52% (51%-53%) | 33% (33%-34%) |
| Percent difference | -7% | -3% | -15% | -16% | -23% |
|  |  |  |  |  |  |
| Percent of staff that are people of color | 33% (32%-34%) | 72% (71%-73%) | 44% (43%-45%) | 51% (50%-51%) | 37% (37%-37%) |
| Percent of supervisors or higher that are people of color | 28% (26%-29%) | 68% (65%-70%) | 33% (31%-35%) | 40% (39%-41%) | 31% (31%-32%) |
| Percent difference | -15% | -6% | -25% | -22% | -16% |

Note: Shown as Estimate (95% Confidence Interval)

Appendix Figure 1: Percent of staff by supervisory status, by gender and race/ethnicity in 2014 & 2017 (pooled)

Appendix Figure 2: Perceptions of workplace environment


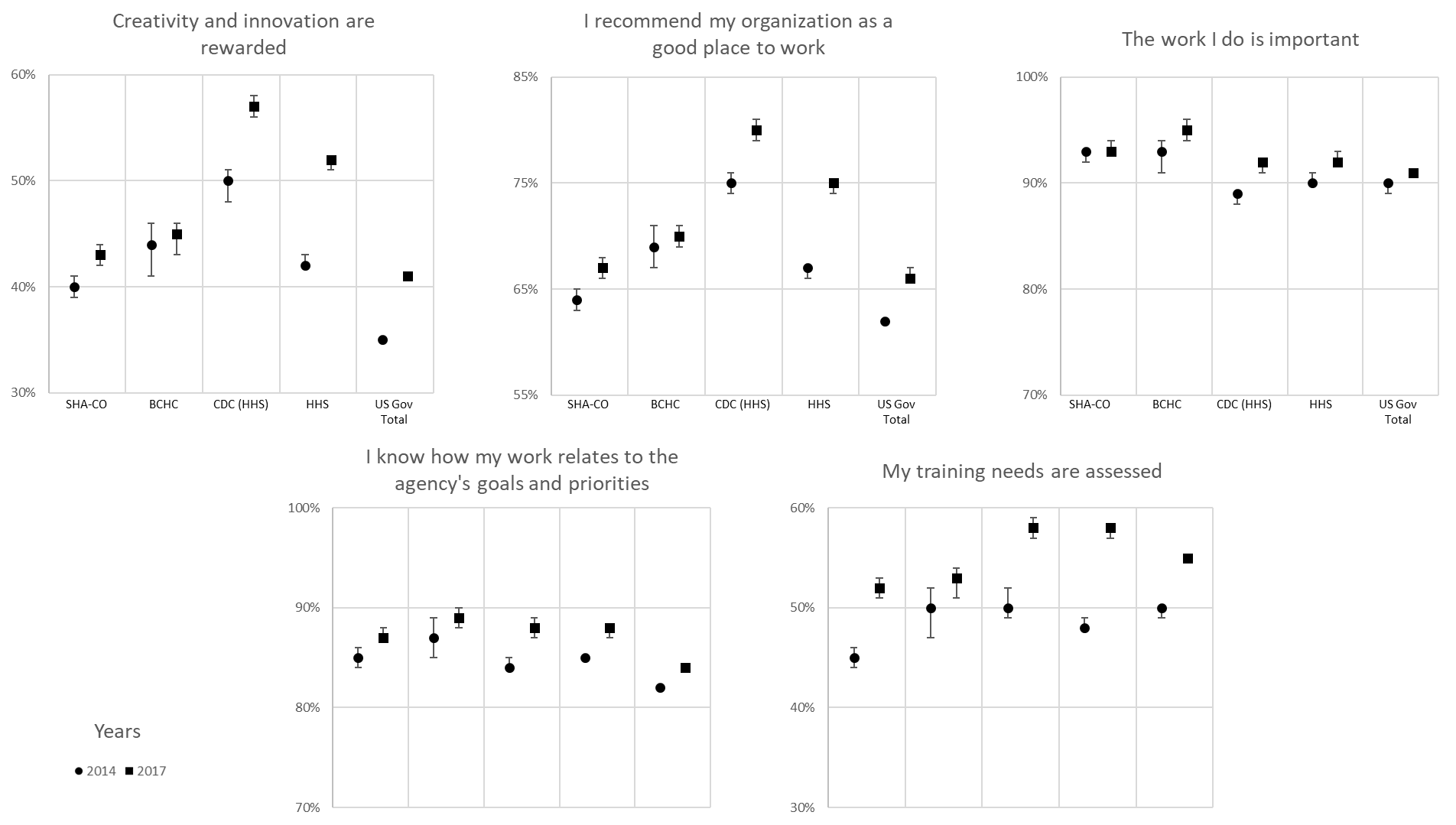


Appendix Figure 3: Agree/strongly agree with statement: Supervisors work well with employees of different backgrounds


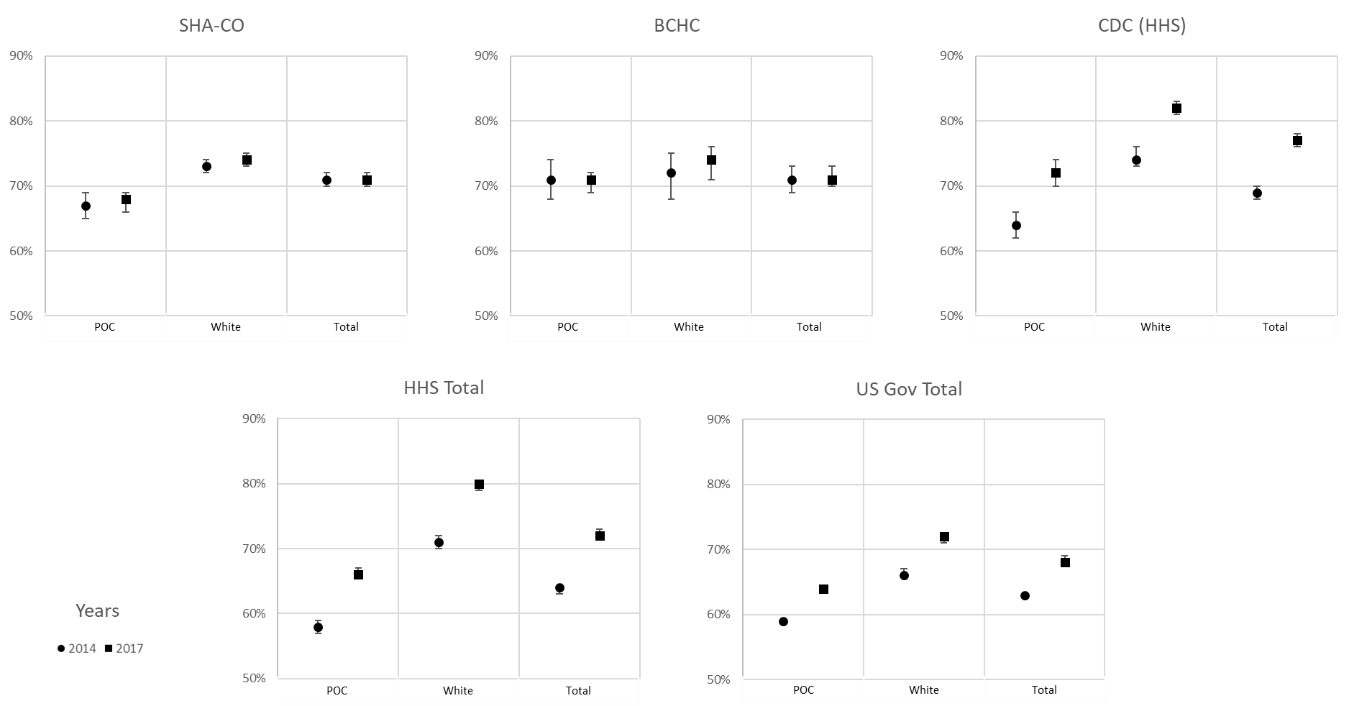


Note: POC – Person of color; White – non-Hispanic White

SHA-CO – State Health Agency Central office; BCHC – Big City Health Department; CDC – Centers for Disease Control and Prevention; HHS – Health and Human Services; EPA – Environmental Production Administration; US Gov total – all federal employees of US government.

Bars are 95% confidence interval
